# Prevalence and predictive factors of complementary medicine use during the first wave of the COVID-19 pandemic of 2020 in the Netherlands

**DOI:** 10.1186/s12906-022-03528-x

**Published:** 2022-02-15

**Authors:** Louise T. C. Mulder, Martine Busch, Agnete E. Kristoffersen, Johanna Hök Nordberg, Esther T. van der Werf

**Affiliations:** 1 Louis Bolk Institute, Kosterijland 3-5, 3981 AJ Bunnik, The Netherlands; 2 Van Praag Institute, Springweg 7, 3511 VH Utrecht, The Netherlands; 3 Dutch Consortium for Integrative Medicine and Health (CIZG), Utrecht, The Netherlands; 4 National Research Centre in Complementary and Alternative Medicine (NAFKAM), Department of Community Medicine, UiT The Arctic University of Norway, Tromsø, Norway; 5 Regional Cancer Centre Stockholm Gotland, Stockholm, Sweden; 6 Department Neurobiology, Care Sciences & Society, Division of Nursing & Department Physiology & Pharmacology, Karolinska Institutet, Stockholm, Sweden

**Keywords:** Prevalence, CM provider, Self-management strategies, Self-care techniques, Survey, The Netherlands

## Abstract

**Introduction:**

Major life changing events such as the COVID-19 pandemic may have major impact on one’s health and general well-being. This study aimed to determine the prevalence and predictive factors, including gender specific differences, of Complementary Medicine (CM) use (including CM consultations, self-care management and self-help techniques) during the first wave of the COVID-19 pandemic in 2020 in the Netherlands.

**Methods:**

CM use was studied among a random representative sample (*n* = 1004) of the adult Dutch population using an online survey conducted from 22–27 May 2020. The survey included a modified version of I-CAM-Q and additional questions on demographic characteristics, reasons for CM use, perceived effectiveness and side effects.

**Results:**

68.0% of the participants reported to have used CM (CM consultations (13.3%), self-management strategies (59.4%), self-help techniques (30.0%)). Most frequently reported reason of CM use was to improve general well-being (61.6%), prevention and/or treatment of COVID-19 was only reported by 10%. Perceived effectiveness of CM was high and number of experienced side effects low. Being a women, worried to get infected with COVID-19, higher education and living in northern/ middle region of the Netherlands were predictive factors to use CM.

**Conclusions:**

In the Netherlands, specific groups (e.g. women/ highly educated) use CM, mainly to improve general wellbeing, and seem to benefit of it during the first months of the pandemic. The high perceived effectiveness and low reporting of side effects should encourage medical professionals and policy makers for more openness towards considering CM as being part of an integrative approach to public health in times life changing events occur.

**Supplementary Information:**

The online version contains supplementary material available at 10.1186/s12906-022-03528-x.

## Background

Worldwide, the self-reported prevalence of any type of complementary medicine (CM) use is varying, ranging from 10 to 76% [1, 2], partly due to the different definitions of CM used. To illustrate, previous study reported estimated use of CM, including consultations and self-care, in the US (34%) [3], Australia (66%) [4] and Eastern Asian countries (over 50%) [5], as well as in several European countries as Italy (14%) [6], Norway (62%) [7], Switzerland (57%) [8] and Germany (70%) [9]. Respectable data on CM use in the Netherlands is limited to CM provider consultations which has been estimated on 11% [10].

Large variety in CM definitions is existing mainly due to differences in healthcare systems, geographical location and culture [11]. Besides, multiple terms are also being used for defining CM such as traditional medicine (TM), complementary medicine (CM), traditional and complementary medicine (T&CM), integrative medicine (IM), or complementary and alternative medicine (CAM) [12]. In general, CM is defined as a group of diverse medical and health care symptoms, practices and products that are not generally considered part of conventional medicine [11].

The three most commonly used CM therapies in Europe are massage therapy, homeopathy and osteopathy [13]. CM is frequently used with the expectation on influencing the natural history of the disease; being in control of one’s health; to manage and relieve symptoms, experience fewer side effects and also for illness prevention and/or boosting the immune system [14–16]. The prevalence of CM use is substantially higher in specific clinical populations such as patients in the oncology field (51%) [17], psychiatry (43%) [18, 19] or children (30%) [20].

It is known that injury/illnesses cause negative impact on both affective and cognitive well-being [21]. However, not only (serious) illnesses but also other major life changing events, defined as occurrences (social, psychological and environmental) which require an adjustment or effect a change in an individual’s pattern of living [22], might greatly influence one’s general well-being.

An example of such a life changing event causing radical changes in the lives of the Dutch population is the (intelligent) lockdown from 15^th^ March till 1^st^ of July 2020 which was enforced by the Dutch government during the first wave of the COVID-19 pandemic [23]. The Dutch population experienced considerable levels of stress and concerns during the first weeks due to the pandemic, especially concerns about their current health state [24]. The possible relationship between CM use and life changing events has not been broadly examined yet. However, a previous study reported that patients with chronic pain who were already using CM therapies, during a major life event increase their seek for CM and seek even more different forms of CM [25].

In general, CM users seem to be more health oriented and show a healthier lifestyle behaviour compared to non CM users [26, 27]. CM use is believed to be closely associated with socio demographic variables such as gender, age, education and income [13, 28, 29]. Especially the association with higher CM use and being a women, highly educated and having a higher age has been well established in literature [10, 13, 30–32]. It would be of interest to determine if these findings hold during life changing events.

Therefore, the aim of this study is to determine the prevalence and predictive factors, including gender specific differences, of overall CM use, CM consultations, self-care management and self-help techniques of the Dutch population during the first wave of the COVID-19 pandemic.

## Methods

The current study reports on Dutch data derived from an international cross-sectional survey on CM use and self-care strategies for prevention and treatment of COVID-19 related symptoms, carried out in Norway, Sweden and the Netherlands in spring 2020 [33].

Data was derived via an online survey in collaboration with Ipsos Netherlands, performed between May 22 and May 27 2020. An internal Ipsos tool has been used to gather the respondents. The respondents registered into the Ipsos Interactive Services (IIS) panel have shared their baseline information such as age, gender, region, and more specific information on education, income and work [33, 34]. From the panel of 45,000 Dutch residents, a representative sample of 4611 (based on the baseline parameters) was invited to complete the survey until 1,000 responses were received. Individuals who were reached and refused participation (*n* = 3,607) were considered non-respondents, leading to a response rate of 22%. The final sample contained 1,013 individuals.

This online survey consisted of a modified version of the International Questionnaire to Measure Use of Complementary and Alternative Medicine (I-CAM-Q) [35]. The modified I-CAM-Q consisted of four parts with two additional categories “for prevention of COVID-19” and “to treat COVID-19-related symptoms” were added to the reasons for use options.

The survey included questions about visits to conventional and CM health care providers (see table 2 for complete list of providers), self-management strategies such as use of natural remedies (see table 3 for complete list of natural remedies/ (food) supplements), and self-help techniques such as mindfulness (see table 4 for complete list of self-help techniques) used within 3 months prior to survey completion. For this study CM use is defined as all treatments and (self) care strategies that are used in addition or as an alternative to the usual (regular) care of e.g. general practitioner, medical specialist, dietician, physiotherapist or nurse in the past 3 months. Perceived effectiveness is defined as to what extent a particular CM use was effective in the perception of the user and ought not to be confused with the actual effectiveness of a certain CM use/treatment. The questions regarding specific CM therapies were adapted to the Netherlands (See supplementary material, Additional file 1).

Demographic characteristics collected were gender, region of residence, age, living environment, living situation, yearly household income, and highest completed level of education. Yearly household income was categorised as low (< EUR 25,000), middle (EUR 25,000 – 74,999), or high (≥ EUR 75,000). Level of education was grouped in three categories; lower education (no school/primary school only/lower secondary education), secondary education (middle and higher secondary education) and higher education ((applied) university/ post-doctoral level). Age was obtained as an open question and assessed as four categories (18–30 years; 31–50 years; 51–65 and 65 years or over).

Three additional questions were posed with regards to worries about COVID-19, rated on a scale from 1–5, where 1 is the lowest and 5 the highest: 1) How worried are you of becoming infected with the virus causing COVID-19 disease?; 2) How worried are you that some of your friends and family could become infected with the virus causing COVID-19 disease?; and 3) Do you think COVID-19 is more dangerous than ordinary influenza?. The continuous variables 1–5 were in the analyses merged into three categorical variables: Not worried (1,2), somewhat worried (3) and very worried (4,5).

Taking into account multiple response biases, the survey was designed as followed: 1) answer options were randomized, meaning every participant will see the same answer options, but in different order, preventing primacy bias (to decrease the amount of times one answer can be chosen which might lead to survey results being too unfairly weighted towards one option), and 2) questions were formulated in a neutral way when asked about education level, salary, age and gender to prevent prestige/stereotype bias as much as possible. Respondents received a personal link (password/username) to prevent filling in the survey more than once and to prevent self-selection bias.

All data was anonymously collected and reported. The anonymous nature of the web-survey did not allow tracing sensitive personal data. The study protocol was reviewed by the Medical Ethical Reviewing Committee of Wageningen University. They decided that this study did not fall within the remit of the Dutch Medical Research Involving Human Subjects Act (WMO), and therefore was exempt from further medical ethical review. Informed consent was obtained from all participants and all participants agreed their data to be used for scientific publication. GDPR guidelines were taken into account [36]. Once completed, each survey was transmitted to the survey platform, and the final database was downloaded.

### Statistical analysis

Descriptive statistics like measures of central tendencies, frequencies and proportions were used to evaluate the responses. Data are represented as number and/or percentage for categorical variables. Pearson’s Chi-square test was performed to identify differences in socio-demographics (age, education level, household income), as well as to identify differences in CM use (general CM use, CM consultation, self-management strategies and self-help techniques) between sexes.

Univariable and multivariable logistic regression was used to identify the (sociodemographic) factors independently associated with CM use in general and CM consultation, self-management strategies and self-help techniques specifically. Multivariable models were derived through several iterations using backward stepwise logistic regression, including all variables that were statistically significant in the univariable analyses.

Statistics were carried out using Statistical Package for Social Sciences (SPSS) v. 26.0. Results were statistically significant for *p* value < 0.05.

## Results

A total of 1013 individuals completed the online questionnaire, and after validation of the data, 1004 respondents (age 18–88 years) were included in the study. Table 1 shows the baseline characteristics and attitude towards COVID-19 of our study population, including 509 female respondents (50.7%). Approximately one third (31.9%) of the respondents were 31–50 years old. Around half of the population had completed a high level of education (49.9%) and was categorized to have a middle income (49.7%). Married respondents living with or without children represented 63.3% of the sample. Of all respondents, 83.1% considered COVID-19 more dangerous compared to the normal influenza virus and 19.1% was very worried to get infected themselves. One third (32.6%) of the respondents reported to be very worried for a close family member or friend getting infected, with women indicating more often to be worried than men (X^2^ = 13.20;*p* = 0.001).

**Table 1:** Baseline and socio-demographic characteristics by gender (*n* = 1004)

	**Total population**	**Gender**	
	*n* = 1004	Male *n* = 495 (49.3%)	Female *n* = 509 (50.7%)
	n (%)	n (%)	n (%)
**Age category**			
18–30	192 (19.1)	89 (18.0)	103 (20.2)
31–50	320 (31.9)	158 (31.9)	162 (31.8)
51–65	267 (26.6)	130 (26.3)	137 (26.9)
65 +	225 (22.4)	118 (23.8)	107 (21.0)
**Education**			
Lower Education	167 (16.6)	75 (15.2)	92 (18.1)
Secondary Education	336 (33.5)	182 (36.8)	154 (30.3)
Higher Education	501 (49.9)	238 (48.1)	263 (51.7)
**Region**			
Northern Regions	274 (27.3)	131 (26.5)	143 (28.1)
Central Regions	277 (27.6)	140 (28.3)	137 (26.9)
Southern Regions	453 (45.1)	224 (45.3)	229 (45.0)
**Living environment**			
Urban	467 (46.5)	239 (48.3)	228 (44.8)
Sub-urban	239 (23.8)	109 (22.0)	130 (25.5)
Rural/Sub-rural	298 (29.7)	147 (29.7)	151 (29.7)
**Living situation**			
Married/living together (without children)	386 (38.4)	195 (39.4)	191 (37.5)
Married/living together (with children)	250 (24.9)	127 (25.7)	123 (24.2)
Living alone without children	249 (24.8)	117 (23.6)	132 (25.9)
Living alone with children	33 (3.3)	10 (2.0)	23 (4.5)
Living with (grand)parents/family	73 (7.3)	39 (7.9)	34 (6.7)
Student accommodation	13 (1.3)	7 (1.4)	6 (1.2)
**Yearly income**^**a**^			
Lower income	150 (14.9)	55 (11.1)	95 (18.7)
Middle income	499 (49.7)	287 (58.0)	212 (41.7)
Higher income	146 (14.5)	78 (15.8)	68 (13.4)
Prefer not to say	209 (20.8)	75 (15.2)	134 (26.3)
**Worries with regards to COVID-19**			
*To get infected yourself*			
Not	390 (38.8)	208 (42.0)	182 (35.8)
Somewhat	422 (42.0)	205 (41.4)	217 (42.6)
Very	192 (19.1)	82 (16.6)	110 (21.6)
*Close family/friend infected*^a^			
Not	205 (20.4)	123 (24.8)	82 (16.1)
Somewhat	472 (47.0)	228 (46.1)	244 (47.9)
Very	327 (32.6)	144 (29.1)	183 (36.0)
*Danger of COVID-19 in comparison with normal influenza virus*			
Less dangerous	14 (1.4)	8 (1.6)	6 (1.2)
Evenly dangerous	156 (15.5)	86 (17.4)	70 (13.8)
More dangerous	834 (83.1)	401 (81.0)	433 (85.1)
**CM use**			
General CM use^b^	683 (68.0)	289 (58.4)	394 (77.4)*
CM provider consultation	134 (13.3)	53 (10.7)	81 (15.9)*
Self-management strategies	596 (59.4)	252 (50.9)	344 (67.6)*
Homeopathic remedies	102 (10.2)	35 (7.1)	67 (13.2)*
Bach flowers	41 (4.1)	15 (3.0)	26 (5.1)
Herbal medicine	191 (19.0)	73 (14.7)	118 (23.2)*
Vitamins/minerals	552 (55.0)	229 (46.3)	323 (63.5)*
Other CM^c^	141 (14.0)	64 (12.9)	77 (15.1)
Self-help techniques	301 (30.0)	107 (21.6)	194 (38.1)*

^*^ Statistically significantly different between sex with *p* < 0.05

^a^ Division of categories is statistically significantly different between sex

^b^ Included when at least one CM mode (consultation, self-management strategies or self-help techniques) has been used

^c^ Including omega 3, 6, 9; Co-enzyme Q10; Protein drink/shake; Probiotics; Glucosamine-chondroitin-MSM

### CM use

Table 1 shows that 68.0% of the total study population reported to have used CM, meaning that they either had consulted a CM provider, made use of self-management strategies or self-help techniques, during the first three months of the COVID-19 pandemic. Women made statistically more use of (all modes) of CM (77.4%) compared to men (58.4%). Most frequently used were self-management strategies (59.4%), followed by self-help techniques (30.0%). A minority (13.3%) reported to have consulted a CM provider. In general, 61.6% of the respondents reported to have used CM to improve general well-being, only 10.0% did this for COVID-19 prevention and/or treatment.

### CM provider consultations

During the first three months of the COVID-19 pandemic women more often consulted a CM provider (15.9%) compared to men (10.7%). The main reasons for consulting was to improve general well-being. Only 2.8% (*n* = 5) consulted a CM provider specifically with the intention to prevent or treat COVID-19 infection, such as a (foot)reflexologist (*n* = 2). The most frequently consulted CM providers were the massage therapist (6.1%), chiropractor (2.6%) and acupuncturist (1.7%). The massage therapist was mainly consulted for complaints in the musculoskeletal system such as back complaints. Chiropractors (76.9%) were most frequently consulted for the treatment of chronic illness or complaints. Most respondents (76.3%) perceived their consult as very effective. A total of 17.2% experienced side effects of their consult (See Additional file 2: Table 2).

### Self-management strategies

Additional file 3: Table 3 shows the use of self-management strategies during the first three months of the COVID-19 pandemic. Vitamins/minerals were the most frequently used self-management strategy and used by more than half of the study population (55.0%). Women used more homeopathic remedies (13.2% vs 7.1%), herbal medicine (23.2% vs. 14.7%) and vitamins and minerals (63.5% vs 46.3%) compared to men. Vitamin D was the most frequently used (23.5%), followed by multivitamins (19.1%). Main reasons reported for use of Vitamin D were lack of sunlight, recommended by a doctor for aging related symptoms and prevention of osteoporosis. Multivitamins, normal and high dose of vitamin C were mainly used to boost resistance and to prevent common colds.

In general, self-management strategies were used by 61,0% to improve general well-being. Only 4.6% (*n* = 77) of the respondents indicated this with the specific intention to prevent or treat COVID-19 infection, such as vitamin C (high dose (*n* = 16); usual dosage (*n* = 13)) and vitamin D (*n* = 9). Homeopathic remedies were used for all kinds of reasons: acute illness (30.4%), chronic illness (28.4%) or complaints and general well-being (55.9%). Of the respondents who reported to use Echinacea, 25.6% indicated their use to treat an acute illness or complaints. Calcium (30.6%), magnesium (18.2%) and zinc (18.2%) were mostly used to treat chronic illness and complaints. Overall, all self-management strategies were perceived to be very effective. A minimal number of respondents experienced side effects.

### Self-help techniques

Around one third (30.0%) of the respondents reported to have used self-help techniques during the first three months of the COVID-19 pandemic, women (38.1%) statistically significantly more compared to men (21.6%). Meditation/mindfulness (10.8%), yoga (10.8%) and relaxation techniques (10.0%) were the most frequently used self-help techniques. Women used these three self-help techniques alongside making music or painting more often than men. Other self-help techniques reported were sports or any sort of physical activity, being in nature such as walking, cycling or gardening.

The main reason to use self-help techniques was to improve the general well-being (74.6%). Only 3.4% (*n* = 18) indicated to have used self-help techniques with the specific intention to treat or prevent COVID-19, with half of this group (*n* = 9) reporting to do this by praying (for their own health) to prevent COVID-19 infection. Of the respondents (*n* = 82) who indicated to pray for their own health, 70.7% did this on a daily basis. Overall, respondents perceived the self-help techniques to be very effective, and only less than 5 participants indicated to have experienced side effects (See Additional file 4: Table 4).

### Predictive factors of CM use

Additional file 5: Table 5 shows the univariable statistically significant associated variables with CM provider consultations, use of self-management strategies and use of self-help techniques during the first wave of the COVID-19 pandemic in the Netherlands that are entered into the multivariable analyses to come to the final models (*P* < 0.05). Based on univariable analyses, gender, region and worried to get infected with COVID-19 were statistically associated with CM provider consultations. For self-management strategies significant associations were found with gender, age, education, region and worries to get infected with COVID-19 themselves or a close family member/friend. With respect to self-help techniques, significant associations were found with gender, age, education, and worries about a family member or friend getting infected with COVID-19.

The final multivariable model (See Additional file 5; Table 5) included 1004/1004 (100%) of the respondents of the survey. Two predictors were strongly associated with CM provider consultations: gender (OR: 1.54, 95% C.I. 1.06 – 2.23) and worries getting infected with COVID-19 (OR *not:* 1.00, OR *somewhat*: 0.94, 95% C.I. 0.61 – 1.44; OR *very*: 1.73, 95% C.I. 1.08 – 2.77). Together these gave an AUROC of 0.59 (95% C.I. 0.54–0.65).

Four predictors were strongly associated with use of self-management strategies: gender (OR: 1.99, 95% C.I. 1.36 – 2.59), education (OR *low* 1.00, OR *middle*: 1.48, 95% C.I. 1.01 – 2.18; OR *high*: 1.61, 95% C.I. 1.12 – 2.33), region (OR *north*: 1.00; OR *middle*: 0.82, 95% C.I. 0.57 – 1.16; OR *south*: 0.64, 95% C.I. 0.47 – 0.89) and worries getting infected with COVID-19 (OR *not:* 1.00, OR *somewhat*: 1.04, 95% C.I. 0.78 – 1.38; OR *very*: 1.80, 95% C.I. 1.24 – 2.63). Together these gave an AUROC of 0.64 (95% C.I. 0.60–0.67).

Three predictors were associated with the use of self-help techniques: gender (OR 2.13, 95% C.I. 1.60 – 2.84), age (OR *18–30*: 1.00, OR *31–50*: 0.59, 95% C.I. 0.41 – 0.88; OR *51–65*: 0.45, 95% C.I. 0.30 – 0.68; OR *65* + 0.29, 95% C.I. 0.18 – 0.47) and worries (OR *not:* 1.00, OR *somewhat*: 1.02, 95% C.I. 0.74 – 1.41; OR *very*: 1.80, 95% C.I. 1.21 – 2.67). Together these gave an AUROC of 0.67 (95% C.I. 0.64–0.71).

## Discussion

This study aimed to determine the prevalence and predictive factors, including gender specific differences, of CM use (CM consultations, self-management strategies and self-help techniques) of the Dutch population during the first wave of the COVID-19 pandemic in 2020.

Sixty-eight per cent (68,0%) of the study population reported to have used at least one modality of CM during the first three months of the COVID-19 pandemic, though the prevalence among women was (statistically) significantly higher compared to men in all modalities (77.4% vs 58.4% respectively). In general, self-management strategies (59.4%), especially intake of vitamin/minerals (55.0%), had most often been used, followed by self-help techniques (30.0%) and CM provider consultations (11.4%). CM was mainly used to increase general well-being (61.6%) and, to a lower extent, for treatment of acute or chronic illnesses or complaints. Only 10.0% of our study population reported to have used CM for COVID-19 prevention and/or treatment. Overall, the reported perceived effectiveness of CM use was high and the number of side effects were minimal. Predictive factors for CM use during the first wave of the COVID-19 pandemic were gender, worries to get infected with COVID-19, education, age and region.

Our data revealed massage therapists as the most consulted CM provider (6.1%), followed by chiropractors (2.6%) and acupuncturists (1.7%). These findings with respect to prevalence and sort of CM provider consultations are in line with previous reported data from 2018 showing that 11% of the Dutch population consulted a CM provider [10], most often the osteopath, chiropractor and acupuncturist [10]. This indicates that no substantial increase or different approach regarding CM consultations has occurred due to the pandemic in these first months. Extensive data on prevalence of self-management strategies and self-help techniques of the Dutch population is lacking. However, previous research on the use of food supplements, reported that 57% of the Dutch population used any form of food supplements, with multivitamins, vitamine D, vitamine C, magnesium as the most frequently used [37]. These results are also comparable with our findings.

Vitamin C and D are previously being associated with decreased respiratory infections rates and better recovery of disease [38, 39], and along with the mineral zinc they provide the most support for the immune system [40]. Our respondents reported taking these and other vitamins especially to improve the immune system and to improve their general well-being as well. While at the time of our study it had been suggested that vitamin D could play a role in preventing and/or treating COVID-19 [41–43], we did not find specific vitamin intake with the main reason to prevent or treat COVID-19 related symptoms. In March 2021 the Health Council of the Netherlands evaluated that there was no need for advising an (increased) intake of vitamin D for the prevention of COVID-19 for the general population [44].

Our findings of a strong association between gender, higher education and younger/middle age and CM use are in line with previously described results in literature [10, 13, 29–31]. Overall, women seem to be more health orientated, interested in, and actively seeking health-related information compared to men, also paying more attention to worldwide pandemics [45]. Specifically the younger generation and highly educated seem to be using self-help techniques more often.

While a life changing event such as an infectious disease outbreak could induce acute stress and psychological concerns, it could also have major long-term impact on our overall health and well-being [46]. Due to the pandemic the Dutch population experienced considerable levels of stress and concerns during the first weeks [24]. Previous studies have already shown that mind–body practices, such as yoga and meditation have beneficial effects on mental health and reduce stress in different populations and circumstances [47–51]. A study performed in the early months of the COVID-19 pandemic, reported an increased use of mind–body practices with promoting health, reducing stress and relaxation reported as the three most important reasons [52]. As we found that worries to get infected was an important predictive factor for the use of all CM modalities and 74.6% of the respondents indicated to use self-help techniques including yoga and meditation to promote general well-being, this implies that reducing stress and psychological concerns with respect to COVID-19 could play a role.

Since CM is getting more popular and accepted, there is a need of evidence on the quality, effectiveness and safety of some CM modalities [53]. The majority of CM users in the Netherlands has been satisfied with their CM use [10] and CM use in several specific patient groups with chronic pain [54] or cancer [55] has been perceived as effective, not only with reduced treatment-related side effects but improved quality of life as well. Our study also indicates a positive experience including a high perceived effectiveness and low reporting of side effects of all CM modalities, which indicate that people seem to benefit from their CM use. However, our results on adverse events have been limited by the fact that only frequency data has been gathered.

Some strengths and limitations of this study need to be noted. Our study has been strengthened by the fact that data was collected during the first critical months during the COVID-19 pandemic in the Netherlands. This gives an unique insight in CM use of the general Dutch population, including consulting CM providers, self-management strategies and self-help techniques, during these times. Responses from over 1000 individuals were rapidly collected within a period of five days from a representative sample of the population. The guaranteed 100% anonymity in collecting and reporting of the data the respondents in this survey may have increased the validity of sensitive information such as health care consultations and health use. Another strength is that our sample size was sufficiently large for detecting correlations.

One of the limitations of this study is the rather low response rate of 22% to the survey which could have increased the risk of non-response bias. Since the assessment of CM use was measured via a self-reported questionnaire based on individual recall methods, respondents may have overestimated or underestimated their CM use. Important to mention is the urgency in which data was gathered to assess behaviour changes within the critical first months of the pandemic. Unfortunately, as a result the additional questions to the I-CAM-Q could not have been pilot tested before. Therefore it is not clear whether these questions were fully understandable and acceptable for the target group, and if this could have influenced the flow and clarity of the survey. Additionally, the cross-sectional design of this study does not allow us to derive causal relationships from the results. Lastly, the low prevalence of CM use used to prevent or treat COVID-19 might be due to the rather low number of infected people at the time of the study. It is therefore likely that a survey among people infected with COVID-19 or another time point in the COVID-19 pandemic would have resulted in a different outcome regarding CM use to prevent and treat COVID-19 [34].

In times of a pandemic people are facing risks of adverse health effects due to quarantine measures such as reduced social contact, self-isolation and other restrictions [56]. Interestingly, our study reveals that the Dutch did not use CM specifically for the prevention or treatment of COVID-19, but rather to improve general well-being. Previous research has already demonstrated the complex relationship between the immune system and multiple lifestyle factors such as exercise, stress reduction, healthy diet, surround with nature and well-being [57–60], and therefore it is known that the general population could benefit from strengthening their resilience through simple preventive means and self-care. Most European countries, including the Netherlands, keep silent when it comes to promoting CM practices in prevention or treatment of COVID-19 unless it comes to safety precautions [61]. In the light of the COVID-19 pandemic an integrated approach could play an important role in the general well-being and quality of life of the general population and worldwide [62].

## Conclusion

CM has substantially been used in the Netherlands during the first three months of COVID-19, mainly to improve general well-being. A high perceived effectiveness has been reported and the number of reported side effects are to be neglected. The COVID-19 pandemic has a major health impact on all populations worldwide and therefore, studies like ours are helpful in providing the foundation for the medical profession and policy makers for openness in considering CM as being part of an integrative approach to public health in times life changing events occur.

## Supplementary Information

**Additional file 1.** Survey (Dutch), modified version of the International Questionnaire to Measure Use of Complementary and Alternative Medicine (I-CAM-Q).

**Additional file 2: Table 2.** CM provider consultations with corresponding reasons, perceived effectiveness and side effects.

**Additional file 3: Table 3.** Use of self-management strategies with corresponding reasons, perceived effectiveness and side effects.

**Additional file 4: Table 4.** Use of self-help techniques with corresponding reasons, perceived effectiveness and side effects.

**Additional file 5: Table 5.** Univariate and multivariate logistic regression analyses with significant associated factors and final models for total CM use, CM provider consultations, use of self-management strategies and use of self-help techniques in the past three months (*n *=1004).

## Data Availability

The datasets used and/or analysed during the current study are available from the corresponding author on reasonable request.

## Abbreviations

CM: Complementary Medicine
OR: Odds ratio
CI: Confidence interval
