# Supplementary material for: Prevalence and predictive factors of complementary medicine use during the first wave of the COVID-19 pandemic of 2020 in the Netherlands
Source: BMC Complement Med Ther. 2022 Feb 15;22:43. doi: 10.1186/s12906-022-03528-x (PMC8845358; doi:10.1186/s12906-022-03528-x)
Supplement: Supplementary file 2 — Additional file 2: Table 2. CM provider consultations with corresponding reasons, perceived effectiveness and side effects. [file 12906_2022_3528_MOESM2_ESM.docx]

Table 2 CM provider consultations with corresponding reasons, perceived effectiveness and side effects

|  |  | Reasons^1^ | | | Perceived effectiveness | | | | Experience of side effects | | |
| --- | --- | --- | --- | --- | --- | --- | --- | --- | --- | --- | --- |
|  | **n (%)** | **Treatment acute illness/ complaints**  **(with a duration <1 month)** | **Treatment chronic illness/ symptoms**  **(with a duration**  **>1 month)** | **Improving general well-being** | **Very effective** | **A little effective** | **Not effective** | **I don’t know** | **Yes** | **No** | **I don’t know** |
| Homeopath | 14 (1.4) | 1 (7.1) | 3 (21.4) | 7 (50.0) | 7 (50.0) | 6 (42.9) | - | 1 (7.1) | 2 (14.3) | 11 (78.6) | 1 (7.1) |
| Acupuncturist | 17 (1.7) | 1 (5.9) | 5 (29.4) | 8 (47.1) | 14 (82.4) | 1 (5.9) | 2 (11.8) | - | 3 (17.6) | 14 (82.4) | - |
| Naturopath | 13 (1.3) | 3 (23.1) | 5 (38.5) | 5 (38.5) | 11 (84.6) | 2 (15.4) | - | - | 4 (30.8) | 9 (69.2) | - |
| Anthroposophical health care provider | 5 (0.5) | 3 (60.0) | - | 2 (40.0) | 3 (60.0) | 2 (40.0) | - | - | - | 5 (100.0) | - |
| Osteopath | 15 (1.5) | 2 (13.3) | 6 (40.0) | 6 (40.0) | 11 (73.3) | 2 (13.3) | 1 (6.7) | 1 (6.7) | 3 (20.0) | 11 (73.3) | 1 (6.7) |
| Chiropractor | 26 (2.6) | 1 (3.8) | 20 (76.9) | 5 (19.2) | 21 (80.8) | 5 (19.2) | - | - | 4 (15.4) | 21 (80.8) | 1 (3.8) |
| Energy healer | 9 (0.9) | - | - | 8 (88.9) | 7 (77.8) | 2 (22.2) | - | - | 2 (22.2) | 7 (77.8) | - |
| (Foot) Reflexologist | 7 (0.7) | 1 (14.3) | 1 (14.3) | 2 (28.6) | 5 (71.4) | 1 (14.3) | 1 (14.3) | - | 1 (14.3) | 6 (85.7) | - |
| Massage therapist | 61 (6.1) | 8 (13.1) | 22 (36.1) | 22 (36.1) | 49 (80.3) | 10 (16.4) | 2 (3.3) | - | 9 (14.8) | 48 (78.7) | 4 (6.6) |
| Traditional Chinese Medicine | 2 (0.2) | 1 (50.0) | - | 1 (50.0) | 1 (50.0) | 1 (50.0) | - | - | 1 (50.0) | 1 (50.0) | - |
|  |  |  |  |  |  |  |  |  |  |  |  |
| ^1^ Percentages do not add up to 100% since the respondents could fill in multiple reasons to consult a CM provider | | | | | | | | | | | |
