# Supplementary material for: Prevalence and predictive factors of complementary medicine use during the first wave of the COVID-19 pandemic of 2020 in the Netherlands
Source: BMC Complement Med Ther. 2022 Feb 15;22:43. doi: 10.1186/s12906-022-03528-x (PMC8845358; doi:10.1186/s12906-022-03528-x)
Supplement: Supplementary file 3 — Additional file 3: Table 3. Use of self-management strategies with corresponding reasons, perceived effectiveness and side effects. [file 12906_2022_3528_MOESM3_ESM.docx]

Table 3 Use of self-management strategies with corresponding reasons, perceived effectiveness and side effects

|  |  | | Reasons^1^ | | | Perceived Effectiveness | | | | | Experience of side effects | | |
| --- | --- | --- | --- | --- | --- | --- | --- | --- | --- | --- | --- | --- | --- |
|  | **N (%)** | **Treatment acute illness/ complaints (with a duration**  **<1 month)** | | **Treatment chronic illness/ symptoms**  **(with a duration**  **>1 month)** | **Improving general**  **well-being** | **Very effective** | **A little effective** | **Not effective** | **I don’t know** | **Yes** | | **No** | **I don’t know** |
|  |  |  | |  |  |  |  |  |  |  | |  |  |
| *Bach flowers* | 41 (4.1) | | 5 (12.2) | 6 (14.6) | 21 (51.2) | 26 (63.4) | 5 (12.2) | 5 (12.2) | 5 (12.2) | | 4 (9.8) | 32 (78.0) | 5 (12.2) |
|  |  | |  |  |  |  |  |  |  | |  |  |  |
| *Homeopathic remedies^2^* | 102 (10.2) | | 31 (30.4) | 29 (28.4) | 57 (55.9) | 93 (91.2) | 42 (41.1) | 4 (3.9) | 6 (5.9) | | 8 (7.8) | 132 (13.1) | 5 (4.9) |
|  |  | |  |  |  |  |  |  |  | |  |  |  |
| *Herbal medicine* | | | | | | | | | | | | | |
| Echinacea | 43 (4.3) | | 11 (25.6) | 5 (11.6) | 21 (48.8) | 25 (58.1) | 14 (32.6) | 2 (4.7) | 2 (4.7) | | 1 (2.3) | 40 (93.0) | 2 (4.7) |
| Passiflora | 7 (0.7) | | 1 (14.3) | 2 (28.6) | 3 (42.9) | 3 (42.9) | 4 (57.1) | - | - | | - | 6 (85.7) | 1 (14.3) |
| Curcumin | 81 (8.1) | | 1 (1.2) | 7 (8.6) | 49 (60.5) | 41 (50.6) | 27 (33.3) | 3 (3.7) | 10 (12.3) | | 1 (1.2) | 79 (97.5) | 1 (1.2) |
| Red yeast rice (Xuezhikang) | 18 (1.8) | | - | 5 (27.8) | 9 (50.0) | 11 (61.1) | 6 (33.3) | 1 (5.6) | - | | - | 18 (100.0) | - |
| Milk thistle (Silybum marianum) | 2 (0.2) | | - | 1 (50.0) | 1 (50.0) | 1 (50.0) | 1 (50.0) | - | - | | - | 2 (100.0) | - |
| Ashwagandha (Withania somnifera) | 5 (0.5) | | - | - | 4 (80.0) | 1 (20.0) | 4 (80.0) | - | - | | - | 4 (80.0) | 1 (20.0) |
| Cranberry | 59 (5.9) | | 5 (8.5) | 10 (16.9) | 30 (50.8) | 25 (42.4) | 22 (37.2) | 6 (10.2) | 6 (10.2) | | - | 57 (96.6) | 2 (3.4) |
| Black cohosh | 5 (0.5) | | - | - | 4 (80.0) | 2 (40.0) | 3 (60.0) | - | - | | - | 5 (100.0) | - |
| Ginseng | 22 (2.2) | | - | 4 (18.2) | 13 (59.0) | 9 (40.9) | 12 (54.5) | - | 1 (4.5) | | 1 (4.5) | 20 (90.9) | 1 (4.5) |
| Ginkgo biloba | 6 (0.6) | | - | - | 3 (50.0) | 4 (66.7) | 2 (33.3) | - | - | | - | 6 (100.0) | - |
|  |  | |  |  |  |  |  |  |  | |  |  |  |
| *Vitamins/Minerals* | | | | | | | | | | | | | |
| Multivitamins | 192 (19.1) | | - | 9 (4.7) | 162 (84.4) | 106 (55.2) | 73 (38.0) | 1 (0.5) | 12 (6.3) | | 5 (2.6) | 184 (95.8) | 3 (1.6) |
| High dose vitamin C | 65 (6.5) | | 3 (4.6) | 4 (6.2) | 35 (53.8) | 46 (70.8) | 17 (26.2) | - | 2 (3.1) | | 1 (1.5) | 64 (98.5) | - |
| Vitamin C  (usual dosage) | 112 (11.2) | | 4 (3.6) | 6 (5.4) | 80 (71.4) | 49 (43.8) | 48 (42.9) | 6 (5.4) | 9 (8.0) | | 4 (3.6) | 103 (92.0) | 5 (4.5) |
| Vitamin D | 236 (23.5) | | 6 (2.5) | 40 (16.9) | 145 (61.4) | 160 (67.8) | 54 (22.9) | 1 (0.4) | 21 (8.9) | | 3 (1.3) | 226 (95.8) | 7 (3.0) |
| Vitamin B | 81 (8.1) | | 4 (4.9) | 9 (11.1) | 52 (64.2) | 51 (63.0) | 26 (32.1) | - | 4 (4.9) | | 4 (4.9) | 75 (92.6) | 2 (2.5) |
| Selenium | 6 (0.6) | | - | 1 (16.7) | 3 (50.0) | 4 (66.7) | 1 (16.7) | - | 1 (16.7) | | - | 6 (100.0) | - |
| Zinc | 22 (2.2) | | 1 (4.5) | 4 (18.2) | 14 (63.6) | 15 (68.2) | 7 (31.8) | - | - | | 2 (9.1) | 20 (90.9) | - |
| Iron | 30 (3.0) | | 4 (13.3) | 4 (13.3) | 17 (56.7) | 22 (73.3) | 7 (23.3) | 1 (3.3) | - | | 5 (16.7) | 25 (83.3) | - |
| Magnesium | 126 (12.5) | | 4 (3.2) | 23 (18.2) | 82 (65.0) | 88 (69.8) | 30 (23.8) | 2 (1.6) | 6 (4.8) | | 7 (5.6) | 116 (92.1) | 3 (2.4) |
| Calcium | 49 (4.9) | | 1 (2.0) | 15 (30.6) | 23 (46.9) | 36 (73.5) | 9 (18.4) | - | 4 (8.2) | | 1 (2.0) | 46 (93.9) | 2 (4.1) |
|  |  | |  |  |  |  |  |  |  | |  |  |  |
| *Other CM* | | | | | | | | | | | | | |
| Omega 3, 6, 9 | 53 (5.3) | | 1 (1.9) | 4 (7.5) | 42 (79.2) | 37 (69.8) | 12 (22.6) | - | 4 (7.5) | | 1 (1.9) | 51 (96.2) | 1 (1.9) |
| Co-enzyme Q10 | 15 (1.5) | | - | 3 (20.0) | 11 (73.3) | 8 (53.3) | 5 (33.3) | - | 2 (13.3) | | 1 (6.7) | 14 (93.3) | - |
| Protein drink/shake | 49 (4.9) | | 2 (4.1) | 5 (10.2) | 32 (65.3) | 33 (67.3) | 12 (24.5) | 2 (4.1) | 2 (4.1) | | 3 (6.1) | 43 (87.8) | 3 (6.1) |
| Probiotics | 26 (2.6) | | - | 6 (23.1) | 17 (65.4) | 13 (50.0) | 9 (34.6) | 1 (3.8) | 3 (11.5) | | 1 (3.8) | 23 (88.5) | 2 (7.7) |
| Glucosamine-chondroitin-MSM | 19 (1.9) | | 3 (15.8) | 6 (31.6) | 8 (42.1) | 13 (68.4) | 5 (26.3) | 1 (5.3) | - | | 1 (5.3) | 18 (94.7) | - |
|  | | | | | | | | | | | | | |
| *^1^ Percentages do not add up to 100% since the option ‘other’ is not included in the table; ^2^ Percentages do not add up to 100% since three products/remedies could be mentioned per respondent; numbers refer to the amount of homeopathic remedies used instead of the amount of respondents* | | | | | | | | | | | | | |
