# Supplementary material for: Prevalence and predictive factors of complementary medicine use during the first wave of the COVID-19 pandemic of 2020 in the Netherlands
Source: BMC Complement Med Ther. 2022 Feb 15;22:43. doi: 10.1186/s12906-022-03528-x (PMC8845358; doi:10.1186/s12906-022-03528-x)
Supplement: Supplementary file 4 — Additional file 4: Table 4. Use of self-help techniques with corresponding reasons, perceived effectiveness and side effects. [file 12906_2022_3528_MOESM4_ESM.docx]

Table 4 Use of self-help techniques with corresponding reasons, perceived effectiveness and side effects

|  |  | | Reasons^1^ | | | Perceived Effectiveness | | | | Experience of side effects | | |
| --- | --- | --- | --- | --- | --- | --- | --- | --- | --- | --- | --- | --- |
|  | **N (%)** | **Treatment acute illness/ complaints (with a duration <1 month)** | | **Treatment chronic illness/ symptoms (with a duration >1 month)** | **Improving general well-being** | **Very effective** | **A little effective** | **Not effective** | **I don’t know** | **Yes** | **No** | **I don’t know** |
| Meditation/Mindfulness | 108 (10.8) | | 3 (2.8) | 7 (6.5) | 92 (85.2) | 77 (71.3) | 30 (27.8) | 1 (0.9) | - | 3 (2.8) | 102 (94.4) | 3 (2.8) |
| Yoga | 108 (10.8) | | 2 (1.9) | 5 (4.6) | 92 (85.2) | 70 (64.8) | 33 (30.6) | 2 (1.9) | 3 (2.8) | 3 (2.8) | 103 (95.4) | 2 (1.9) |
| Qigong | 6 (0.6) | | - | - | 5 (83.3) | 5 (83.3) | 1 (16.7) | - | - | - | 6 (100.0) | - |
| Tai Chi | 11 (1.1) | | 1 (9.1) | 2 (18.2) | 5 (45.5) | 10 (90.9) | 1 (9.1) | - | - | 1 (9.1) | 10 (90.9) | - |
| Relaxation techniques | 100 (10.0) | | 3 (3.0) | 13 (13.0) | 77 (77.0) | 68 (68.0) | 31 (31.0) | 1 (1.0) | - | 2 (2.0) | 98 (98.0) | - |
| Visualisation | 24 (2.4) | | - | 3 (12.5) | 19 (79.2) | 19 (79.2) | 4 (16.7) | 1 (0.1) | - | 2 (4.2) | 22 (91.7) | - |
| Prayer for own health | 82 (8.2) | | 4 (4.9) | 10 (12.2) | 34 (41.5) | 66 (80.5) | 9 (11.0) | 1 (1.2) | 6 (7.3) | 4 (4.9) | 74 (90.2) | 4 (4.9) |
| Painting/Music for own health | 56 (5.6) | | - | 3 (5.4) | 44 (78.6) | 39 (69.6) | 16 (28.6) | - | 1 (1.8) | 2 (3.6) | 54 (96.4) | - |
|  |  | |  |  |  |  |  |  |  |  |  |  |
| *^1^ Percentages do not add up to 100% since the option ‘other’ is not included in the table* | | | | | | | | | | | | |
