# Supplementary material for: Prevalence and predictive factors of complementary medicine use during the first wave of the COVID-19 pandemic of 2020 in the Netherlands
Source: BMC Complement Med Ther. 2022 Feb 15;22:43. doi: 10.1186/s12906-022-03528-x (PMC8845358; doi:10.1186/s12906-022-03528-x)
Supplement: Supplementary file 5 — Additional file 5: Table 5. Univariate and multivariate logistic regression analyses with significant associated factors and final models for total CM use, CM provider consultations, use of self-management strategies and use of self-help techniques in the past three months (n =1004). [file 12906_2022_3528_MOESM5_ESM.docx]

Table 5 Univariate and multivariate logistic regression analyses with significant associated factors and final models

|  | Total CM use | | CM provider consultations | | | Self-management strategies | | | Self-help techniques | | |
| --- | --- | --- | --- | --- | --- | --- | --- | --- | --- | --- | --- |
|  | Univariate significant variables  (p <0.05) | Final multivariate model  (AUC: 0.67  (0.63-0.70)) | | Univariate significant variables  (p <0.05) | Final multivariate model  (AUC: 0.59  (0.54-0.65)) | | Univariate significant variables  (p <0.05) | Final multivariate model  (AUC: 0.64  (0.60-0.67)) | | Univariate significant variables  (p <0.05) | Final multivariate model  (AUC: 0.67  (0.64-0.71)) |
|  | **OR (95% CI)** | **OR (95% CI)** | | **OR (95% CI)** | **OR (95% CI)** | | **OR (95% CI)** | **OR (95% CI)** | | **OR (95% CI)** | **OR (95% CI)** |
|  |  |  | |  |  | |  |  | |  |  |
| Gender | 2.44 (1.86 – 3.21) | **2.42 (1.83 – 3.21)** | | 1.56 (1.09 – 2.29) | **1.54 (1.06 – 2.23)** | | 2.01 (1.56 – 2.60) | **1.99 (1.36 – 2.59)** | | 2.23 (1.69 – 2.95) | **2.13 (1.60 – 2.84)** |
|  |  |  | |  |  | |  |  | |  |  |
| Age |  |  | |  |  | |  |  | |  |  |
| 18-30 years |  |  | |  |  | | 1.00 |  | | 1.00 | **1.00** |
| 31-50 years |  |  | |  |  | | 1.15 (0.81 – 1.65) |  | | 0.66 (0.45 – 0.95) | **0.60 (0.41 – 0.88)** |
| 51-65 years |  |  | |  |  | | 1.54 (1.06– 2.25) |  | | 0.50 (0.33 – 0.73) | **0.45 (0.30 – 0.68)** |
| 65+ years |  |  | |  |  | | 1.40 (0.95 – 2.06) |  | | 0.31 (0.20 – 0.48) | **0.29 (0.18 – 0.47)** |
|  |  |  | |  |  | |  |  | |  |  |
| Education^1^ |  |  | |  |  | |  |  | |  |  |
| Low | 1.00 | **1.00** | |  |  | | 1.00 | **1.00** | | 1.00 |  |
| Middle | 1.26 (0.86 – 1.84) | **1.35 (0.90 – 2.02)** | |  |  | | 1.39 (0.95 – 2.01) | **1.48 (1.01 – 2.18)** | | 1.01 (0.66 – 1.55) |  |
| High | 1.69 (1.17 – 2.43) | **1.77 (1.21 – 2.61)** | |  |  | | 1.53 (1.07 – 2.18) | **1.61 (1.12 – 2.33)** | | 1.58 (1.07 – 2.35) |  |
|  |  |  | |  |  | |  |  | |  |  |
| Region |  |  | |  |  | |  |  | |  |  |
| Northern Regions | 1.00 | **1.00** | | 1.00 |  | | 1.00 | **1.00** | |  |  |
| Middle Regions | 0.98 (0.68 – 1.42) | **1.00 (0.68 – 1.47)** | | 1.75 (1.06 – 2.89) |  | | 0.81 (0.57 – 1.14) | **0.82 (0.57 – 1.16)** | |  |  |
| Southern Regions | 0.66 (0.47 – 0.91) | **0.64 (0.46 – 0.90)** | | 1.34 (0.83 – 2.16) |  | | 0.65 (0.48 – 0.89) | **0.64 (0.47 – 0.89)** | |  |  |
|  |  |  | |  |  | |  |  | |  |  |
| Worries with regards to COVID-19 |  |  | |  |  | |  |  | |  |  |
| *To get infected yourself* |  |  | |  |  | |  |  | |  |  |
| Not | 1.00 | **1.00** | | 1.00 | **1.00** | | 1.00 | **1.00** | | 1.00 | **1.00** |
| Somewhat | 0.94 (0.70 – 1.25) | **0.95 (0.70 – 1.29)** | | 0.96 (0.63 – 1.47) | **0.94 (0.61 – 1.44)** | | 1.02 (0.77 – 1.34) | **1.04 (0.78 – 1.38)** | | 0.84 (0.62 – 1.15) | **1.02 (0.74 – 1.41)** |
| Very | 1.94 (1.30 – 2.92) | **2.01 (1.32 – 3.06)** | | 1.80 (1.13 – 2.88) | **1.73 (1.08 – 2.77)** | | 1.77 (1.22 – 2.55) | **1.80 (1.24 – 2.63)** | | 1.40 (0.97 – 2.01) | **1.80 (1.21 – 2.67)** |
| *Close family/friend infected* |  |  | |  |  | |  |  | |  |  |
| Not | 1.00 |  | | 1.00 |  | | 1.00 |  | | 1.00 |  |
| Somewhat | 0.99 (0.70 – 1.39) |  | | 1.35 (0.79 – 2.30) |  | | 1.04 (0.75 – 1.44) |  | | 0.97 (0.67 – 1.40) |  |
| Very | 1.62 (1.11 – 2.37) |  | | 1.83 (1.06 – 3.16) |  | | 1.44 (1.01 – 2.06) |  | | 1.52 (1.04 – 2.23) |  |
|  |  |  | |  |  | |  |  | |  |  |
| *^1^ Lower education (no school/primary school only/lower secondary education), secondary education (middle and higher secondary education) and higher education ((applied) university/ post-doctoral level)* | | | | | | | | | | | |
